# Addition of Cryoprotectant Significantly Alters the Epididymal Sperm Proteome

**DOI:** 10.1371/journal.pone.0152690

**Published:** 2016-03-31

**Authors:** Sung-Jae Yoon, Md Saidur Rahman, Woo-Sung Kwon, Yoo-Jin Park, Myung-Geol Pang

**Affiliations:** Department of Animal Science and Technology, Chung-Ang University, Anseong, Gyeonggi-do, Republic of Korea; Nanjing Medical University, CHINA

## Abstract

Although cryopreservation has been developed and optimized over the past decades, it causes various stresses, including cold shock, osmotic stress, and ice crystal formation, thereby reducing fertility. During cryopreservation, addition of cryoprotective agent (CPA) is crucial for protecting spermatozoa from freezing damage. However, the intrinsic toxicity and osmotic stress induced by CPA cause damage to spermatozoa. To identify the effects of CPA addition during cryopreservation, we assessed the motility (%), motion kinematics, capacitation status, and viability of epididymal spermatozoa using computer-assisted sperm analysis and Hoechst 33258/chlortetracycline fluorescence staining. Moreover, the effects of CPA addition were also demonstrated at the proteome level using two-dimensional electrophoresis. Our results demonstrated that CPA addition significantly reduced sperm motility (%), curvilinear velocity, viability (%), and non-capacitated spermatozoa, whereas straightness and acrosome-reacted spermatozoa increased significantly (p < 0.05). Ten proteins were differentially expressed (two decreased and eight increased) (>3 fold, p < 0.05) after CPA, whereas NADH dehydrogenase flavoprotein 2, f-actin-capping protein subunit beta, superoxide dismutase 2, and outer dense fiber protein 2 were associated with several important signaling pathways (p < 0.05). The present study provides a mechanistic basis for specific cryostresses and potential markers of CPA-induced stress. Therefore, these might provide information about the development of safe biomaterials for cryopreservation and basic ground for sperm cryopreservation.

## Introduction

Cryopreservation is a long-term storage technique for preserving cells, embryos, or tissues without damage induced by chemical reactivity or time [1, 2]. It is a valuable technique for storing cells with a limited life span and reducing the risk of microbial contamination, cross contamination with other cell lines, genetic drift, and morphological changes [3, 4]. Cryopreservation has a number of clinical and research applications, such as assisted reproductive techniques, genetic improvement, management of degreases, and biobanking [5, 6].

In general, sperm cryopreservation is performed on ejaculated semen. However, in several cases such as valuable deceased males, unexpected death, and catastrophic injury, cryopreservation of epididymal sperm play an important role to reserve genetic information [7, 8]. After death of an animal, spermatozoa in the testis are alive for a period of time. Therefore, storing these spermatozoa using cryopreservation and subsequent in vitro fertilization (IVF) can be useful tools for rescuing genetic resources [9].

Sperm cryopreservation contributes to the development of reproductive techniques, such as artificial insemination (AI) and *in vitro* fertilization (IVF) [10, 11]. Frozen semen allows for the management of selection and breeding in domestic animals, resulting in advances of the livestock industry [12, 13]. Moreover, it is an important tool for genome resource banking for species conservation [14]. However, cryopreservation inevitably causes various types of stress, such as cold shock, osmotic stress, and ice crystal formation, thereby reducing fertility [15]. Although cryopreservation has been developed and optimized over the past decades, the process still causes up to a 50% loss of viable spermatozoa [16].

Cryopreservation has three steps: dilution with the extender/cooling, addition of cryoprotective agent (CPA), and freeze-thawing [17]. These processes cause various types of stresses, such as cold shock, osmotic and oxidative stress, and intracellular ice crystal formation subsequently affect membrane structures, organelle functions, and fertility [17].

Among these processes, the addition of CPA step plays a crucial role in cryopreservation [15]. To protect spermatozoa from freezing damage, such as ice crystal formation, CPA is added during cryopreservation [18]. As a standard CPA, glycerol causes rearrangement of membrane proteins and lipid of spermatozoa. These actions increase membrane fluidity and dehydration thereby reduce intracellular ice crystal formation in spermatozoa [19, 20]. Simultaneously, addition of CPA induced massive osmotic stress to spermatozoa, which is the major factor of cryoinjury [19]. It has been reported that CPA is capable to penetrate plasma membrane rapidly and alters the sperm head volume, finally resulting in the damage to membrane surface [21]. Spermatozoa are sensitive to toxic effect of CPA and components of the sperm membrane can damage by toxicity of CPA [22, 23]. Moreover, the addition of CPA induces osmotic stress and excessive reactive oxygen species (ROS) generation, resulting in disruption of mitochondrial membrane potential, alteration of membrane permeability, and damage of sperm surface proteins [21, 24].

It is well known that changes in protein composition, through protein degradation and/or post-translational modifications (such as phosphorylation), can affect sperm function during cryopreservation [21, 24]. Moreover, Sorrenti *et al*. [25] have reported on the conformational changes in proteins that were associated with membrane function, motility, viability, and fertilizing ability.

Several studies have compared spermatozoa before and after cryopreservation in various species [12, 26]. However, to the best of our knowledge, there has been no study to identify the effects of CPA addition at the proteome level thus far. Here, in order to investigate the effects of CPA addition on the sperm proteome, a comprehensive proteomic study was performed using bovine spermatozoa as a potential *in vitro* model.

## Material and Methods

### Ethical statement

All animal procedures were followed the guidelines for the ethical treatment of animals. All processes of animal treatments were approved by the Institutional Animal Care and Use Committee of Chung-Ang University, Seoul, Korea.

### Sample collection

All procedures followed by methods of Yoon *et al*. [17]. We obtained testes of Native Korean Bull (Hanwoo) from a slaughterhouse (Seomun Co., Hwaseong, Korea). Bulls were fed according to typical Korean Feeding Standard for Native Korean Bull (Hanwoo), which is consisted of three phases, i.e. First phase (6 to 14 months): 4 kg of commercial concentrate per day and roughage composed of 0.5 kg of alfalfa hay, 1.2 kg of timothy, and tall fescue hay per day; Second phase (15 to 22 months): 7.7 kg of commercial concentrate per day with free access to rice straw; Third phase (22 to 31 months): 7.7 kg of concentrate with 0.8 kg of rice straw per day. The testes were transferred to the laboratory within 3 h with ice [27]. Sperm samples were collected from nine individual bull epididymides using back flushing with phosphate-buffered saline (PBS; Sigma-Aldrich, St. Louis, MO, USA). Collected samples in 15mL tube were washed at 700 ×*g* for 15 min [28].

### Sperm treatment

To investigate the effects of CPA addition during cryopreservation, sperm cryopreservation with routine concentration of CPA was performed as previously described, with some modifications [17, 29]. Briefly, collected samples (fresh sperm) were resuspeneded (100 × 10^6^ cells/mL) in Tris–egg yolk buffer (TYB; 250 mM Tris, 88.5 mM citric acid, 68.8 mM glucose, and 20% egg yolk) and cooled from room temperature (RT) to 4°C over 2 h. Sample was diluted in an equal volume 12% glycerol TYB (final concentration was 6%). Then, sample was equilibrated at 4°C for 2 h.

### Computer-assisted sperm analysis (CASA)

Sperm motility (%) and the kinematics of samples before and after CPA addition were analyzed with a computer-assisted sperm analysis (CASA) system (Sperm Analysis Imaging System version SAIS-PLUS 10.1; Medical Supply, Seoul, Korea) as previously described [17]. Briefly, 10 μL of each sample was placed on a preheated (37°C) Makler chamber (Makler, Haifa, Israel). The obtained images from 10× objective in phase contrast mode were analyzed with the SAIS software. At least 250 sperm cells was evaluated for sperm motility (%) and kinematics, such as curvilinear velocity (VCL), straight-line velocity (VSL), average path velocity (VAP), straightness (STR), amplitude of lateral head displacement (ALH), beat-cross frequency (BCF), linearity (LIN) and wobble (WOB).

### Assessment of capacitation status and viability

Capacitation status and viability were determined using an H33258/CTC dual staining method, as previously described [17, 30]. Briefly, 15 μL of H33258 solution (10 μg H33258/mL PBS) was added to 135 μL of the sample. The mixed sample was incubated for 10 min at RT. To remove excess dye, 250 μL of 2% (w/v) polyvinylpyrrolidone (Sigma-Aldrich) in PBS was added to the mixture and washed at 700 ×*g* for 5 min. The pellet was resuspended in 150 μL of PBS and 150 μL of CTC solution (750 mM CTC in 5 μL of buffer composed of 20 mM Tris, 130 mM NaCl, and 5 mM cysteine, pH 7.4). To assess viability and capacitation status, a Microphot-FXA microscope (Nikon, Tokyo, Japan) under epifluorescent illumination was used with ultraviolet BP 340–380/LP 425 filters for H33258 and BP 450–490/LP 515 filters. Four different patterns were observed: live acrosome-reacted (AR pattern: sperm showing mottled green fluorescence over the head, green fluorescence only in the post-acrosomal region, or no fluorescence over the head), live capacitated (B pattern: green fluorescence over the acrosomal region and a dark post-acrosomal region), live non-capacitated (F pattern: green fluorescence distributed uniformly over the entire sperm head, with or without a stronger fluorescent line at the equatorial segment), or dead (D pattern: blue fluorescence distributed uniformly over the entire sperm head). Capacitation status and viability were observed at least 400 spermatozoa per slide.

### 2DE, gel-image analysis, and protein identification

2DE was performed to find differentially expressed proteins during step of CPA addition. All procedures followed by methods of Kwon *et al*. [31]. To rule out the individual difference on cryoprotectant related stress, nine bivine sperm samples were mixed for proteomic analysis. Sperm samples of before and after CPA addition were washed by centrifugation at 700 × *g* for 15 min on isotonic 45% Percoll in PBS [13, 31].

After rehydration with 250 μg solubilized protein sample for 12 h at 4°C, 24-cm-long NL Immobiline DryStrips (pH 3–11; Amersham, Piscataway, NJ, USA) were focused for first-dimension electrophoresis using an IPGphor isoelectric focusing device. After equilibration of the strips, 2-DE was performed with 12.5% (w/v) SDS-polyacrylamide gel electrophoresis (SDS-PAGE) gels. Silver staining was performed and the each gel was scanned with a GS-800 calibrated scanner (Bio-Rad, Hercules, CA, USA). To quantify, detection, and matching spots, PDQuest 8.0 software (Bio-Rad, Hercules, CA, USA) was used. All procedures repeated three times in each sample treatment (n = 9). Also identification procedures followed by methods of Kwon *et al*. [31]. After trypsin digestion, the peptides were desalted and concentrated. The peptide analyzation was performed by nano-electrospray ionization on a Q-TOF2 mass spectrometer (AB Sciex Instruments). The protein data was obtained from the ion search option in the MASCOT software (Matrix Science).

### Signaling pathway

To find out signaling pathways associated with protein markers, Pathway Studio (v 9.0, Aridane Genomics, Rockville, MD, USA) was used. Differentially expressed proteins were inputted in Pathway Studio to determine significantly matched pathways for each protein.

### Western blotting

Western blotting was performed to confirm the 2-DE results as described previously [31], with modifications. Commercial polyclonal anti-SOD2 (Abcam, Cambridge, MA, USA) and polyclonal anti-NUDFV2 (Abcam) were used, and monoclonal anti-α-tubulin (Abcam) was used as a control. Briefly, the sperm samples of before and after CPA addition were washed two times with PBS at 10,000 ×*g* for 5 min in RT. The pellets were resuspended with lysis buffer containing 5% 2-mercaptoethanol and incubated for 10 min at RT. The insoluble fractions were separated by centrifugation at 10,000 ×*g* for 10 min. The samples were electrophoresed on a 12% SDS-polyacrylamide gel. Then sample transferred to polyvinylidene fluoride membranes (Amersham). The membranes were blocked with PBS-Tween containing 5% skim milk powder (blocking solution) for 3 h at RT. After washing, the membranes were incubated overnight with anti-NDUFV2 (1:3,000) and anti-SOD2 (1:5,000) diluted with blocking solution. Then, the membranes were incubated with a horse radish peroxidase (HRP) conjugated anti-rabbit immunoglobulin G (IgG) (1:3000, Abcam) for 1 h. After the membranes were washed, proteins were detected by enhanced chemiluminescence reagents. All bands were scanned using a GS-800 Calibrated Imaging Densitometer (Bio-Rad) and analyzed with Quantity One (Bio-Rad). The signal intensity ratios of the bands were calculated for SOD and NUDFV2 normalized to α-tubulin. All procedures repeated three times in each sample treatment (n = 9).

### Statistical analysis

Data were analyzed with SPSS v.21.0 (SPSS Inc., Chicago, IL, USA). A Student’s two-tailed *t*-test was used to compare values for before and after adding CPA, after performing normality and variance homogeneity tests. p < 0.05 was considered statistically significant. Data are expressed as mean ± SEM. Fisher’s exact test was used to determine the probability that a protein is involved in a particular signaling pathway (p < 0.05).

## Results

### Sperm parameters

**Fig 1:**
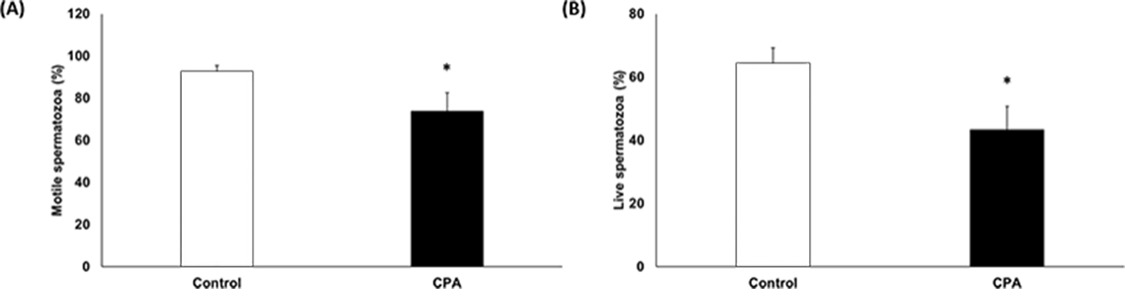
Effects of CPA addition on sperm (**A**) motility (**B**) viability. Data are presented as the mean ± SEM. (*p < 0.05, n = 9).

**Fig 2:**
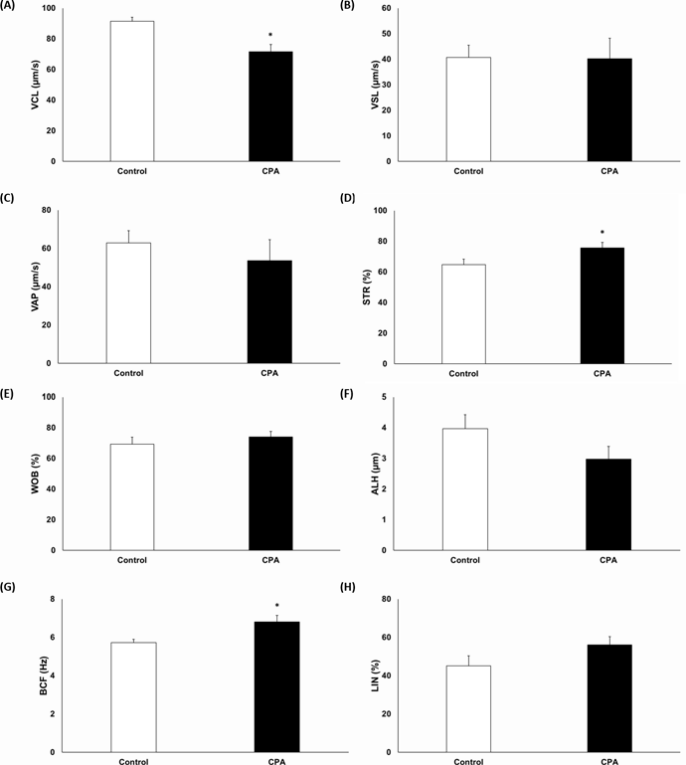
Effects of CPA addition on sperm motion kinematics. (**A**) Curvilinear velocity, (**B**) straight-line velocity, (**C**) average path velocity, (**D**) straightness, (**E**) wobble, (**F**) amplitude of lateral head, (**G**) beat-cross frequency, and (**H**) linearity. Data are presented as mean ± SEM. (*p < 0.05, n = 9).

**Fig 3:**
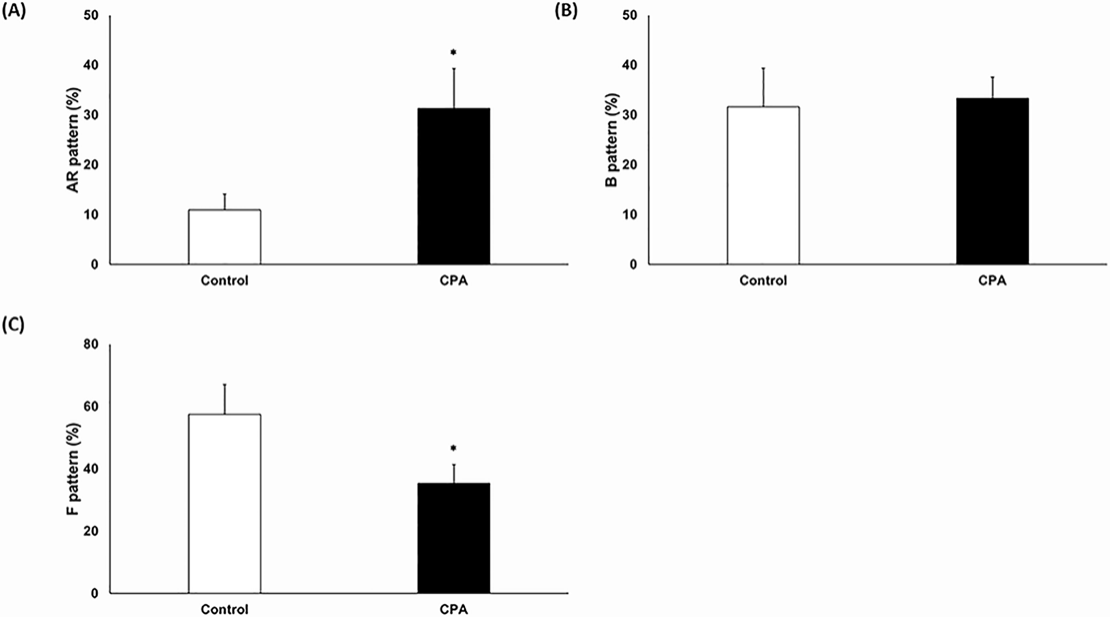
Effects of CPA addition on live sperm capacitation status as assessed by combined H33258 /CTC staining (**A**) AR pattern, (**B**) B pattern, (**C**) F pattern. Data are presented as the mean ± SEM. (*p < 0.05, n = 9).

Motility and VCL were significantly decreased after CPA addition (Figs 1 and 2, p < 0.05); conversely, STR and BCF were significantly increased (Fig 2, p < 0.05). However, there were no significant differences in VSL, VAP, WOB, ALH, and LIN (Fig 2). Sperm capacitation status and viability were evaluated using H33258/CTC duel staining. The AR pattern was significantly increased, whereas the F pattern was significantly decreased after CPA addition (Fig 3, p < 0.05). However, there were no differences for the B pattern (Fig 3). Viability of spermatozoa after CPA addition was significantly lower than for fresh spermatozoa (Fig 1, p < 0.05).

### 2-DE

**Fig 4:**
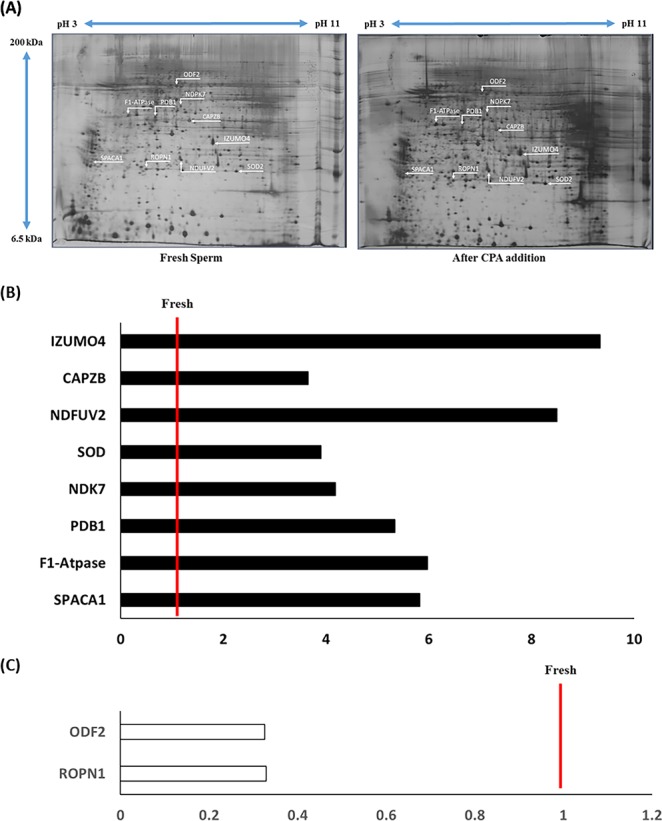
Representative 2-DE gel image of protein separation. Protein spots from (**A**) fresh spermatozoa and after CPA addition. (**B**) The expression of eight proteins increased significantly after CPA addition. (**C**) The expression of two proteins decreased significantly after CPA addition. Differentially expressed (>3-fold) proteins were determined by comparing the normalized spot value of particular protein between the fresh and samples after CPA addition (*P < 0.05, n = 9). The red line indicates the landmark of equal levels for fresh spermatozoa. Each experiment was conducted three times.

2-DE was performed with fresh spermatozoa and spermatozoa after CPA addition. A total of 285 proteins were detected, and 10 proteins were differentially expressed (>3 fold, Table 1 and Fig 4). Sperm acrosome membrane-associated protein 1 (SPACA1), chain D, bovine mitochondrial F1-atpase complexed with aurovertin B (F1-ATPase), pyruvate dehydrogenase E1 component subunit beta (PDB1), mitochondrial precursor nucleoside diphosphate kinase 7 (NDPK7), NADH dehydrogenase flavoprotein 2 (NDUFV2), f-actin-capping protein subunit beta (CAPZB), izumo sperm-egg fusion protein 4 (IZUMO4) and superoxide dismutase 2 (SOD2) were highly expressed after the addition of CPA. However, outer dense fiber protein 2 (ODF2) and Ropporin 1 (ROPN1) were highly expressed in the fresh spermatozoa (Table 1 and Fig 4).

**Table 1:** Differentially expressed (> 3-fold) proteins of spermatozoa between fresh spermatozoa and spermatozoa after CPA addition.

Spot no.	Protein	NCBI no.	^a^MASCOT score	Change in level
223	sperm acrosome membrane-associated protein 1 (SPACA1)	114053081	43.0	increase
1304	chain D, bovine mitochondrial F1-atpase complexed with aurovertin B (F1-ATPase)	1827812	183.0	increase
2207	Ropporin-1 (ROPN1)	115495919	63.0	decrease
3415	pyruvate dehydrogenase E1 component subunit beta, mitochondrial precursor (PDB1)	164420789	109.0	increase
4418	nucleoside diphosphate kinase 7 (NDPK7)	62751773	64.0	increase
4607	outer dense fiber protein 2 (ODF2)	84000345	290.0	decrease
5204	NADH dehydrogenase flavoprotein 2 (NDUFV2)	27807025	67.0	increase
5305	f-actin-capping protein subunit beta (CAPZB)	28603770	53.0	increase
6201	Izumo sperm-egg fusion protein 4 (IZUMO4)	156120505	143	increase
7206	superoxide dismutase 2, mitochondrial (SOD2)	88853816	74.0	increase

^a^MASCOT score is −10 log (P), where P is the probability that the observed match is a random event. Individual scores > 40 indicate identity or extensive homology (p<0.05).

### Western blotting

**Fig 5:**
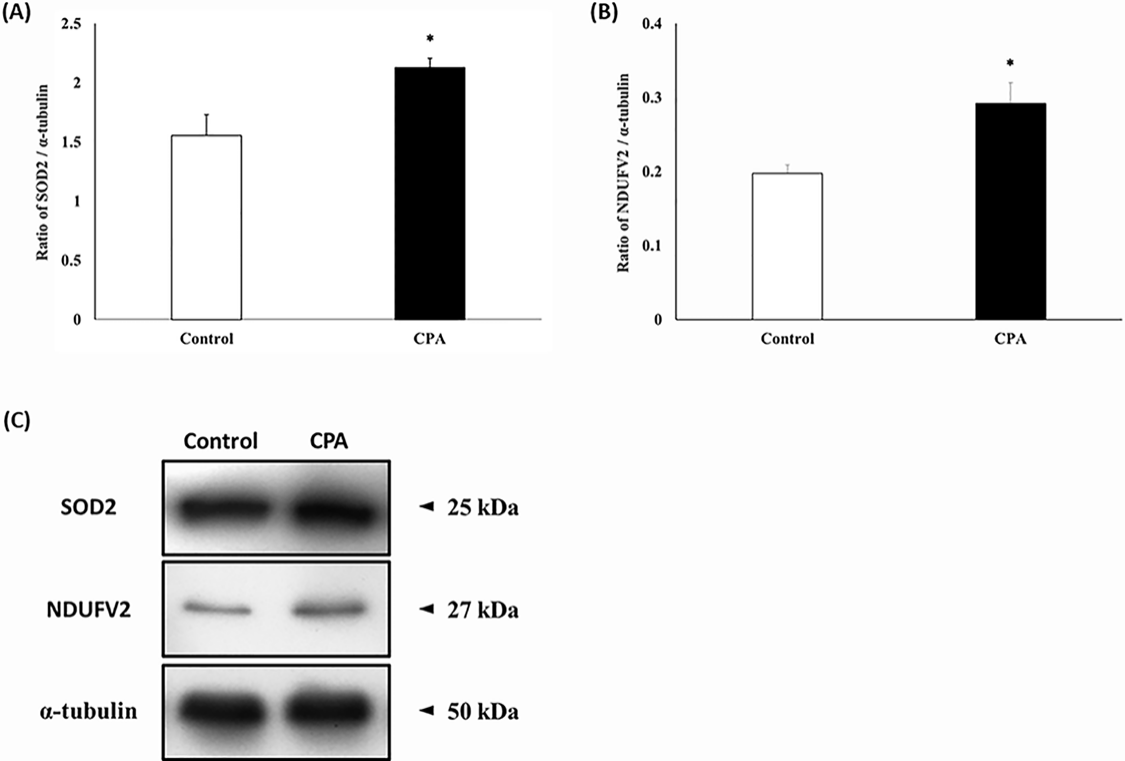
Expression of SOD and NDUFV2 in fresh spermatozoa and spermatozoa after CPA addition. (**A**) The ratio of SOD2 and α-tubulin in fresh samples and samples after CPA addition. (**B**) The ratio of NDUFV2 and α-tubulin in fresh samples and samples after CPA addition. Data are presented as the mean ± SEM. (*p < 0.05, n = 9).

To confirm the 2-DE results, western blotting analysis was performed using commercial antibodies against two differentially expressed proteins (SOD2 and NDUFV2). SOD2 and NDUFV2 were detected at 25 and 27 kDa, respectively. The densities of SOD2 and NDUFV2 were highly increased after CPA addition (Fig 5, p < 0.05), and these results show a similar pattern to the 2-DE results.

### Informatics

Ten differentially expressed proteins from fresh spermatozoa and spermatozoa after CPA addition were imported and analyzed using Pathway studio 9. Five signaling pathways were significantly correlated with four proteins (S1 Fig and Table 2, p < 0.05). CAPZB and ODF2 were correlated with the ephrinR-actin signaling pathway, and SOD2 was correlated with ROS metabolism (Table 2, p < 0.05). The actin cytoskeleton assembly pathway was correlated with CAPZB, and actin cytoskeleton regulation was correlated with CAPZB and ODF2 (Table 2, p < 0.05). Moreover, the respiratory chain and oxidative phosphorylation pathway was correlated with NDUFV2 (Table 2, p < 0.05).

**Table 2:** Signaling pathways associated with differentially expressed proteins as identified by Pathway Studio.

Signaling Pathways	Overlapping Entities	p-value
EphrinR-actin signaling	CAPZB, ODF2	0.030
ROS metabolism	SOD2	0.041
Actin Cytoskeleton Assembly	CAPZB	0.016
Actin Cytoskeleton Regulation	CAPZB, ODF2	0.043
Respiratory chain and oxidative phosphorylation	NDUFV2	0.045

## Discussion

Differences between ejaculated and epididymal spermatozoa were already reported in numerous studies [32, 33]. Fertility of epididymal spermatozoa tends to be lower than ejaculated spermatozoa. In addition, sperm maturity and freezing sensitivity are also different [8, 34]. However, cryopreservation of epididymal spermatozoa can be advantageous for sudden death of animals and catastrophic injured animals because it has capability of fertilization [32, 34, 35]. Moreover, epididymal spermatozoa can be stored at 5°C for 24 hours without loss of sperm motility and fertility [36]. Especially, when animals die unexpectedly at the location far from the laboratory, cryopreservation of epididymal spermatozoa has key role of preserving important genetic resources [9, 36, 37]. In present study we evaluated bovine epididymal spermatozoa before and after addition of CPA to find out general markers of cryo-stress related with CPA.

During cryopreservation, the process of CPA addition reduces the physical and chemical stresses induced by cooling, freezing, and thawing [18, 21]. However, CPA exhibits toxicity that damages spermatozoa. Furthermore, CPA addition causes osmotic stress, change in volume of spermatozoa, sperm membrane destabilization, and protein denaturation [38]. Consequently, various sperm parameters, such as motility, viability, and capacitation status are changed which are parameters are closely related with sperm fertility [21, 24, 39].

Motility and motion kinematics are major factors in fertility. Therefore, these parameters play an essential role for transport of sperm to the fertilization site and contact with the egg [40]. CPA can cause oxidative damage to membrane phospholipids of spermatozoa, resulting in a loss of sperm motility and viability [8, 17]. In our study, motility and VCL were significantly decreased after CPA addition (Figs 1 and 2). On the other hand, STR and BCF were significantly increased after CPA addition (Fig 2). Our study also shows that viability was significantly decreased after CPA addition (Fig 1).

Sperm membrane composition and fluidity are affected by CPA addition and result in loss of membrane-selective permeability [41]. CPA causes an efflux of membrane cholesterol and calcium (Ca^2+^) influx [42]. Ca^2+^ influx triggers capacitation and the acrosome reaction [21]. However, this is also observed in damaged sperm membranes, i.e. nonfunctional capacitation or premature capacitated spermatozoa. Premature capacitation induces an aberrant acrosome reaction in spermatozoa [21, 42]. In the current study, the AR pattern was significantly increased without increased B pattern, and the F pattern was significantly decreased after CPA addition (Fig 3). This suggests that premature AR in spermatozoa is induced by CPA addition.

It has been reported that CPA affects sperm parameters as well as sperm membrane composition [22, 23, 43]. In fact, membrane cytoskeletal components are sensitive to the toxic effect of CPA and it could induce damages of surface proteins such as f-actin [21, 39]. Moreover, toxicity of CPA induces alteration of sperm membrane components [43]. For these reasons, spermatozoa after CPA addition should be examined at the proteome level. Therefore, to elucidate the underlying mechanisms of the effects of CPA addition, a comprehensive sperm proteomic study was performed.

Application of proteomic technique enabled to discover biomarkers related to sperm function [44]. Over the past decades, several new techniques also has been introduced and each of them have different degree of sensitivity and accuracy [45, 46]. Some of these techniques (e. g. ion intensities MS and isobaric labeling) provide higher accuracy, however most of them (e. g. spectrum count MS, APEX,emPAI, metabolic labeling, and isotopic labeling) have low quantative accuracy, even their coverage were satisfactory. On the other hand, 2DE-MS used in the current study provide considerable performance in respect of their moderate coverage and quantative accuracy [45]. Moreover, several studies also considered this technique to study the sperm proteome [47, 48].

Numerous studies have reported that transcription, translation, and protein synthesis do not occur in mature spermatozoa [44]. However, several studies have reported that mature spermatozoa have the capability of protein synthesis via post-translational protein modifications such as phosphorylation [31]. Phosphorylation has an important role in cellular mechanism and regulation of sperm functions [49]. Moreover, several researches suggest that there is a different mechanism involved in cryopreservation, besides protein degradation [31, 50]. We found ten proteins that were differentially expressed after CPA addition (Table 1). Among them, only two proteins (ODF2 and ROPN1) decreased, and eight proteins (IZUMO4, CAPZB, NDUFV2, SOD, NDK7, PDB1, F1-Atpase, and SPACA1) increased after CPA addition (Fig 4).

ODF2 is a cytoskeletal structural protein that is localized in sperm flagella, and it associates with sperm structures and motility [51]. Lack of ODF leads to abnormal morphology and infertility [52]. ODF2 was decreased after CPA addition, and correspondingly, motility and viability were also decreased in the present study (Figs 1 and 4).

ROPN1 is a component of the fibrous sheath of mammalian spermatozoa, and it localizes to the principal piece and the end piece of sperm flagella [53]. ROPN1 is a cyclic adenosine monophosphate-dependent protein kinase regulator, and is related to sperm movement [54]. The spermatozoa from asthenozoospermic patients show a lower expression of ROPN1 than normal spermatozoa [54]. Expression of ROPN1 and motility were significantly decreased after CPA addition in the current study (Figs 1 and 4).

IZUMO4 is a member of IZUMO family of proteins, which is expressed in the testis and is present on mature spermatozoa [55]. IZUMO is essential for gamete fusion, and is exposed on the sperm surface after acrosome reaction [56]. In our study, IZUMO4 was significantly increased after CPA addition. We believe that it might be associated with the increased AR pattern observed in this study (Figs 3 and 4).

CAPZB is a member of the F-actin capping protein family and is a major cytoskeletal protein [57]. CAPZB is involved in F-actin function (38). F-actin depolymerization inhibits capacitation and the acrosome reaction [58]. Capping proteins assemble and disassemble filaments in the outer acrosomal membrane during capacitation, and these mechanisms are important for the induction of the acrosome reaction [59]. Higher expression of CAPZB after CPA addition might be related to the increased AR pattern observed in this study (Figs 3 and 4).

SOD2 is an important antioxidant that dismutates O^2−^ into H_2_O_2_ [60]. It is possible to improve cell survival by reducing the level of ROS [61]. In the testis, SOD2 reacts with ROS directly and alleviates ROS toxicity [62]. CPA causes oxidative stress, which damages spermatozoa during cryopreservation [8]. In our study, SOD2 was highly expressed after CPA addition (Fig 4). Therefore, it is plausible to suggest that this increased expression of SOD2 reflects a defensive response to protect the spermatozoa against oxidative stress [63].

NDPK7 is a member of the NDPK family proteins and is an enzyme that synthesizes nucleoside triphosphates and localizes to mitochondria [64]. NDPK catalyzes reversible reactions of nucleoside triphosphate at the expense of ATP [65]. NDPK is also widely known to regulate transcription, cell proliferation, and energy metabolism [66]. Moreover, it has protective effects against oxidative stress [67]. Cryostress, such as oxidative stress induced by CPA addition, causes metabolic and functional changes in spermatozoa [25]. In our study, NDPK7 increased after CPA addition (Fig 4). This suggests that oxidative stress and changes in energy metabolism are induced by CPA addition in spermatozoa.

PDB1 is one of seven subunits of the pyruvate dehydrogenase complex family, and it catalyzes the oxidative decarboxylation of pyruvate to acetyl CoA [31]. PDB1 localizes in the mitochondrial matrix and is involved in sperm capacitation and the acrosome reaction [68]. In this study, increased PDB1 expression might be related to the increased AR pattern observed after CPA addition (Figs 3 and 4).

Four of the ten differentially expressed proteins resulting from CPA addition were significantly correlated with five signaling pathways (Table 2). These signaling pathways were also associated with sperm functions.

The ROS metabolism pathway is associated with various sperm parameters, such as motility, viability, capacitation, and fertility [69]. The respiratory chain and oxidative phosphorylation pathway is associated with energy metabolism of spermatozoa [70]. CPA addition causes oxidative damage and loss of mitochondrial potential [21, 24, 39]. These types of damage are closely related to ROS and energy metabolism, and result in changes in motility and viability [70]. Therefore, it is tempting to speculate that these signaling pathways are strongly related to the modified sperm function observed after CPA addition.

The actin cytoskeleton assembly and regulation pathways are associated with sperm capacitation and acrosome reaction [71]. In these pathways, phosphatidylinositol (4, 5)-bisphosphate (PIP2) and phosphatidylinositol (3, 4, 5)-trisphosphate (PIP3) are important regulators of actin polymerization [72]. Actin polymerization occurs during capacitation and the acrosome reaction[73]. Actin organization of the ephrinR-actin signaling pathway is also related to the actin cytoskeleton assembly and regulation pathways (S1 Fig). Moreover, PIP2 and PIP3 are associated with the regulation of motility and the cytoskeleton of the membrane [73]. These mechanisms might result in the changes in sperm parameters that are observed in the present study (Fig 1).

In this study, we examined the altered sperm parameters and proteome after CPA addition using bovine epididymal spermatozoa. We also discovered the underlying mechanism of sperm damage by CPA addition at the proteome level as well as the associated signaling pathways. To the best of our knowledge, this is the first study to evaluate the effects of CPA addition during sperm cryopreservation at the proteome level. We provide a valuable tool for identifying the mechanisms of a specific type of cryodamage as well as useful potential markers of damage from CPA addition. This approach can offer a logical ground for sperm cryopreservation and a new way of developing safe biomaterials for cryopreservation.

## Supporting Information

S1 Fig Signaling pathways associated with differentially expressed proteins, as identified by Pathway Studio. (**A**) The ephrinR-actin signaling pathway, (**B**) the ROS metabolism pathway, (**C**) the actin cytoskeleton regulation pathway, (**D**) the actin cytoskeleton assembly pathway, and (**E**) the respiratory chain and oxidative phosphorylation pathway.
(TIF)
