# Supplementary figures and images for: Addition of Cryoprotectant Significantly Alters the Epididymal Sperm Proteome
Source: PLoS One. 2016 Mar 31;11(3):e0152690. doi: 10.1371/journal.pone.0152690 (PMC4816509; doi:10.1371/journal.pone.0152690)

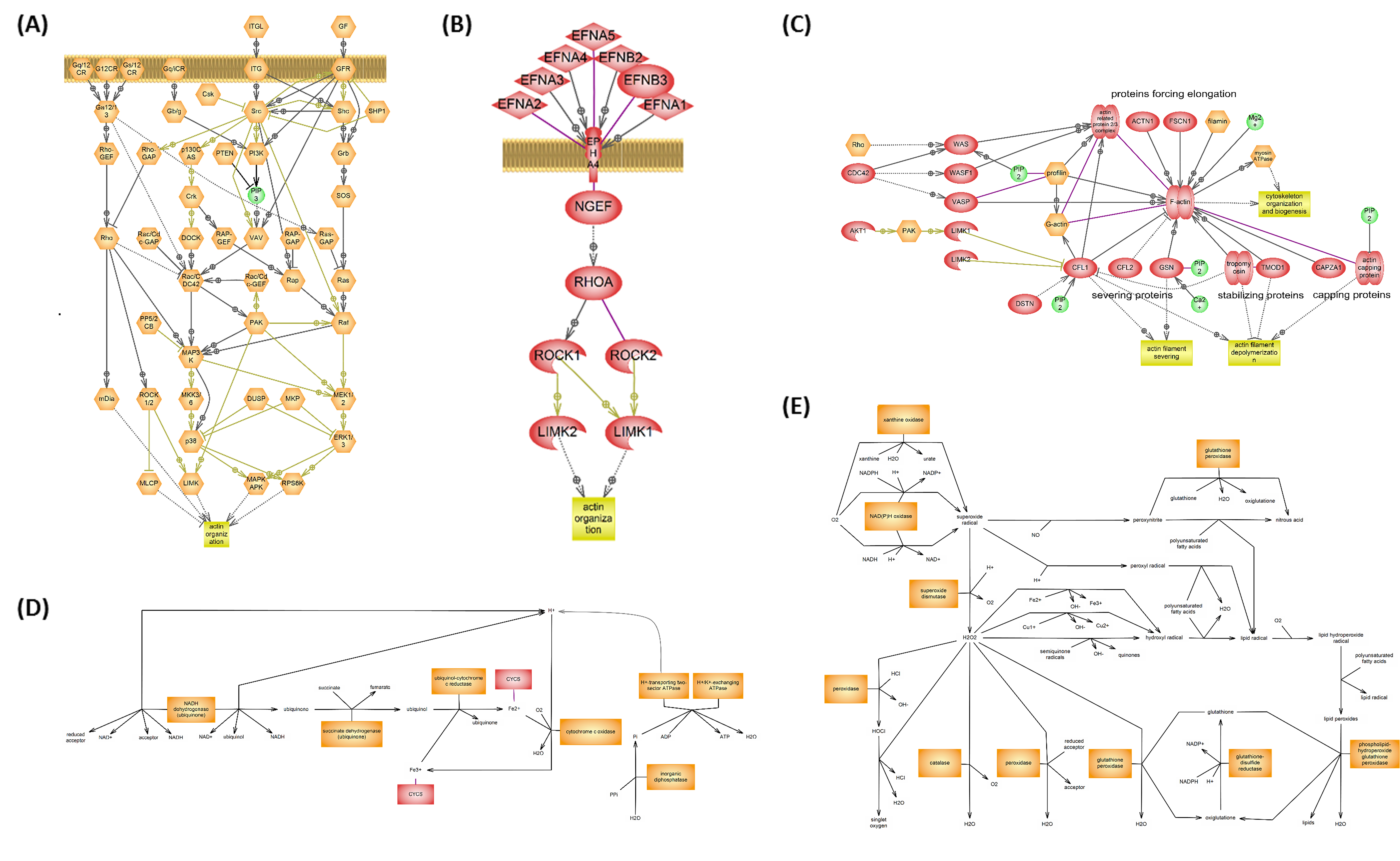

Supplement: S1 Fig — (A) The ephrinR-actin signaling pathway, (B) the ROS metabolism pathway, (C) the actin cytoskeleton regulation pathway, (D) the actin cytoskeleton assembly pathway, and (E) the respiratory chain and oxidative phosphorylation pathway. (TIF) [file pone.0152690.s001.tif]
